# Microenvironment involved in FPR1 expression by human glioblastomas

**DOI:** 10.1007/s11060-015-1777-2

**Published:** 2015-04-19

**Authors:** J. C. Boer, D. M. S. van Marion, J. V. Joseph, N. M. Kliphuis, H. Timmer-Bosscha, J. A. G. van Strijp, E. G. E. de Vries, W. F. A. den Dunnen, F. A. E. Kruyt, A. M. E. Walenkamp

**Affiliations:** Department of Medical Oncology, University Medical Center Groningen, University of Groningen, Hanzeplein 1, P.O. Box 30001, 9700 RB Groningen, The Netherlands; Department of Medical Microbiology, University Medical Center Utrecht, University of Utrecht, Heidelberglaan 100, 3584 CX Utrecht, The Netherlands; Department of Pathology and Medical Biology, University Medical Center Groningen, University of Groningen, Hanzeplein 1, 9700 RB Groningen, The Netherlands

**Keywords:** FPR1, GBM, Mitochondrial peptides, GBM cells

## Abstract

Formyl peptide receptor 1 (FPR1) activity in U87 glioblastoma (GBM) cells contributes to tumor cell motility. The present study aimed to evaluate the FPR1 expression in human GBM, the possibility to elicit agonist induced FPR1 activation of GBM cells and inhibit this activation with chemotaxis inhibitory protein of *Staphylococcus aureus* (CHIPS). Immunohistochemistry was used to assess FPR1 expression in GBM patient samples, which was present in all 178 samples. Also FPR1 mRNA levels measured with quantitative PCR, could be detected in all 25 GBM patient samples tested. Activation of FPR1 in U87 cells, as measured by human mitochondrial-derived agonists, increased calcium mobilization, AKT and ERK1/2 phosphorylation, and ligand-induced migration. Inhibition of all responses could be achieved with CHIPS. Eight early passage human Groningen Glioma (GG) cell lines, isolated from primary GBM tissue were screened for the presence of FPR1. FPR1 mRNA and protein expression as well as receptor activation could not be detected in any of these early passage GG cell lines. However FPR1 was present in ex vivo tumors formed by the same GG cell lines after being implanted in mouse brains. FPR1 is highly expressed in human GBM specimens, it can be activated by human mitochondrial-derived agonists in U87 and inhibited with CHIPS. FPR1 cannot be detected in early passage GG cell lines in vitro, however when engrafted in the mouse brain these cells show FPR1 expression. These results suggest a role of the brain microenvironment in FPR1 expression in GBM.

**Electronic supplementary material:**

The online version of this article (doi:10.1007/s11060-015-1777-2) contains supplementary material, which is available to authorized users.

## Introduction

Glioblastoma (GBM) accounts for circa 65 % of malignant gliomas [1, 2]. Treatment options post surgical resection, consist of radiotherapy with concomitant temozolomide resulting in median survival of 12–15 months [3, 4]. The highly infiltrative nature of GBM and its seemingly preordained recurrence contribute to the necessity of developing new treatment options. Inhibition of tumor cell migration could be a therapeutic strategy. In this respect the formyl peptide receptor 1 (FPR1) might be of interest. FPR1 is a G-protein coupled receptor (GPCR) originally identified by its capability to mediate phagocytic leukocyte migration. Agonists for FPR1 are formylated peptides such as the bacterial derived fMLF, and mitochondrial derived fMLKLIV and fMMYALF [5]. Additionally supernatant from necrotic tumor cells may activate FPR1 on U87 cells [6]. Formylated peptide induced activation of phagocytic leukocytes can be inhibited by Chemotaxis Inhibitory Protein of *Staphylococcus aureus* (CHIPS). CHIPS is an immune evasion protein secreted by *S.aureus* [7] and a selective inhibitor of FPR1 which potently abrogates the migration of neutrophils and monocytes towards the site of infection [8].

Stimulation of human U87 GBM cells with fMLF elicits the upregulation of hypoxia inducible factor 1-alpha (HIF1α) [6] and of vascular endothelial growth factor (VEGF) [6, 9]. Moreover FPR1 receptor activation produces downstream protein phosphorylation of ERK1/2 and AKT, which are early signalling events of cell proliferation and migration [10, 11]. In addition these effects can be inhibited by CHIPS [9]. Furthermore, CHIPS treatment showed modest but improved survival of mice with subcutaneously implanted U87 xenografts [9].

In this study we investigated FPR1 expression in human GBM. We analyzed if human mitochondrial peptides could lead to activated FPR1 mediated responses by U87 cells and whether CHIPS could inhibit these responses. In addition early passage Groningen Glioma (GG) cells were screened for functional FPR1 expression and presence of FPR1 mRNA. Finally we compared the presence of FPR1 in human GG cell lines cultured in vitro and implanted in mouse brains.

## Materials and methods

### Cells

The human GBM cell line U87 was purchased from the ATCC (HTB-14) and cultured as previously described [9]. GG lines; GG1, GG6, GG7, GG9, GG12, GG13, GG14 and GG16, isolated from eight primary GBM specimens, were kept at low passage numbers and cultured as previously described [12].

### Tissue collection

A total of 178 GBM patient specimens were collected. Of these, 141 samples were formalin fixed paraffin embedded (FFPE)(4 cores per tumor) on tissue micro arrays (TMA). In addition 37 frozen specimens with good quality material were used for cryostaining. For 25 specimens sufficient additional tissue was available for quantitative (q)PCR analysis. The paired diagnostic paraffin tumor tissues from which the GG cell lines were isolated was used for comparative staining. Additional control sections of 3 pneumonia and 1 healthy brain tissue were included. All patient samples were retrieved from the tissue bank at the Department of Pathology at the UMCG and collected between 2005–2012 (FFPE samples) and 1998–2007 (cryosections). Tumor tissues were numerically tagged based on a national coding system. According to Dutch law, no further Institutional review and board approval was required.

From NOD scid gamma mice (NOD.Cg-*Prkdc*^*scid*^ *Il2rg*^*tm1Wjl*^/SzJ)/NSG mice), orthotopically implanted with GG12, GG13, GG14 and GG16 FFPE coronal sections were obtained as previously described [12].

### Ca^2+^ mobilization assay

GPCR activation is measurable by calcium release [13] upon ligand induced receptor activation. Calcium-mobilization by 10^−6^–10^−8^ M fMLKLIV and fMMYALF in U87 cells and 10^−5^ M fMLF in GG cell lines, was performed as previously described [9]. For inhibition experiments, cells pre-incubated with 0.01, 0.1, 1 or 10 μg/mL CHIPS for 15 min at room temperature (RT) were stimulated with 10^−7^ M fMLKLIV and or 10^−6^ M fMMYALF. Stimulation/inhibition was calculated using the following formula: [(MF_sample_–bgF_sample_)/(MF_max_–bgF_max_)] × 100 % in which MF_sample_ = mean fluorescence sample, bgF_sample_ = background fluorescence sample, MF = mean fluorescence with stimulation and bgF = background fluorescence without stimulation [14].

### Quantitative PCR

Total RNA extraction was obtained from 25 GBM samples, and GG1, GG6, GG7, GG9, GG12, GG13, GG14, GG16 cell lines. U87 served as a positive control and a no template control (water) served as negative control. RNA was extracted following RNeasy mini kit guidelines (Qiagen, Venlo, The Netherlands) for the GBM samples or with TRIzol^®^-reagent (Ambion Life Technologies, Blijswijk, The Netherlands) by adding 1 mL of TRIzol^®^ to a 25 cm^2^ (U87 and GG7) or 75 cm^2^ culture flask (all other GG cell lines). TRIzol^®^-treated samples were incubated for 10 min, mixed with 200 μL chloroform/mL TRIzol^®^ and centrifuged each time at 4 °C and 21,300×*g* for 5 min. Samples were incubated with 500 μL isopropyl alcohol per 1 mL TRIzol^®^ for 10 min and centrifuged. RNA pellet was washed with 75 % ethanol, centrifuged, air-dried and quantified using a NanoDrop ND-00-spectrophotometer (Thermo Scientific, Breda, The Netherlands). Following Ambion guidelines total RNA was treated with TURBO DNA-free kit; the quality and integrity were detected by ethidium bromide (Invitrogen) staining on 1.2 % agarose gel. Synthesis of cDNA was performed following iSCRIPT guidelines (Bio-Rad Laboratories, Veenendaal, The Netherlands) and quality was checked with a ladder PCR using glyceraldehyde 3-phosphate dehydrogenase (GAPDH) as a loading control. The qPCR was performed using TaqMan Universal Master Mix (Life Technologies) and measured on ABI PRISM 7900HT real-time sequence detection system (Applied Biosystems, Forster City, CA) in a 384-well reaction plate. Primers from Life Technologies included FPR1 (HS04235429_S1) and GAPDH (Hs02758991_g1). Raw data was extracted with SDS software 2.3 (Applied Biosystems) and averages of threshold cycles (C_T_) were used for calculations of relative expression with the 2^−Δct^ method. GAPDH C_T_ served as background value with cutoff set at CT values of 40 cycles.

### Immunohistochemistry and staining evaluation

FFPE (4 µm) slides were deparaffinized and rehydrated. Endogenous peroxidase was blocked with 0.33 % H_2_O_2_, antigen retrieval was performed with TrisHCl (pH 9)/microwave. Primary FPR1 antibody (ab#150533, Abcam, Cambridge, UK) antibody diluted in 1 % bovine serum albumin (BSA)/phosphate buffered saline, 2.7 mM KCl, 1.8 mM KH_2_PO_4_, 137 mM NaCl, 10.1 mM Na_2_HPO_4_, pH = 7.4 (PBS) was incubated at 4 °C overnight. Secondary, tertiary antibodies (Table 1) and 3,3-diaminobenzidine (DAB) system were applied and slides were dehydrated and coverslipped.

**Table 1:** List of antibodies used

Antibody	Source	Antigen retrieval	Type	Dilutions and conditions
FPR1	Abcam ab150533	Tris–HCl, 15 min MW	Rabbit Polyclonal	1:250; O/N 4 °C
	Abcam ab101659	None	Rabbit Polyclonal	1:250; 1 h RT
CD68	Dako	Tris–HCl, 15 min MW	Mouse Monoclonal	1:100; O/N 4 °C
CD163	Leica	Tris–HCl, 15 min MW	Mouse Monoclonal	1:200; O/N 4 °C
GFAP	Dako	None	Rabbit Polyclonal	1:800; O/N 4 °C

*MW* microwave; *min* minutes; O/N = overnight; *h* hour; *RT* room temperature

Snap frozen tissue (4 µm) sections were mounted on Starfrost^®^ adhesive slides, dried for 20 min, acetone-fixed for 10 min and incubated with primary FPR1 antibody (ab#101659, Abcam) in 1 % BSA/PBS at RT for 1 h. Subsequently, secondary and tertiary antibodies were applied, followed by 3-amino-9-ethylcarbazole (AEC) detection. Slides were mounted with Kaiser’s glycerol-gelatin (Millipore Corporation, Amsterdam, the Netherlands). Omission of primary antibody or an appropriate preparation of IgG served as a negative control. Normal brain and pneumonia tissue samples served as controls for antibody specificity. In pneumonia tissue a high FPR1 positivity on infiltrated neutrophils and bronco-alveolar epithelial cell is expected [15, 16]. In the cerebral cortex a moderate neuron positivity is expected [14].

Immunohistochemical evaluation of TMA was assessed by double blind scoring of slide scans acquired with Aperio ImageScope. Semi-quantitative evaluation was performed by attributing 0 (negative) to 3 (positive) scores. Averages of the 4 cores from both observers representing each patient sample were calculated.

Quantitative evaluation of frozen sections was performed using Olympus WH10-H/22 grid by calculating the percentage of all positive cells counted within the grid in 4 high power fields (400x) randomly selected throughout each tumor slide.

### Western blot

For Western blot analysis U87 cells (1 × 10^6^) were seeded, starved for 24 h and pre-incubated with culture medium or 10 μg/mL CHIPS for 20 min at RT. Subsequently, U87 cells were treated for 5, 15, 30 min with culture medium only or containing, 10^−6^ M fMMYALF or fMLKLIV. Cells were washed twice with 1.5 mL ice cold PBS, lysed with 40 μL mammalian protein extraction reagent (MPER)(Thermo Scientific) including 1:100 protease (Thermo Scientific), 1:100 phosphatase (Thermo Scientific) inhibitors and incubated for 1 h on ice. Immunoblotting for phosphorylated Akt(ser473) and ERK1/2 was performed as previously described [9].

### Migration

Eight μm pore-Corning Transwell^®^ polycarbonate membrane cell culture inserts (Sigma-Aldrich, Zwijndrecht, The Netherlands) were coated and blocked as previously described [9]. Lower wells were loaded with 300 μL of 10^−6^ M fMLF, fMMYALF or fMLKLIV in Dulbecco’s modified Eagle’s medium high glucose (DMEM-H)/0.5 % BSA. Before treatment U87 cells were serum starved for 2 days and subsequent steps were performed in serum-free DMEM-H medium. Next, 6 × 10^5^ U87 cells/mL were incubated for 15 min with control medium or medium with 10 μg/mL CHIPS. Then, 150 µL containing 10^5^ U87 cells were loaded on top of transwell inserts in triplicates. U87 cells migrated towards medium containing 10^−6^ M fMLF, fMMYALF or fMLKLIV for 5 h at 37 °C in 5 % CO_2_ humidified atmosphere. Transwell inserts were cleaned; cells were fixed and counted as previously described [9].

### Immunofluorescence

Paraffin sections of GBM patient specimens (4 µm) were deparaffinized, hydrated in demi water, washed with PBS, blocked with PBS/1 % BSA and stained with anti-FPR1 antibody (ab#150533) concomitant either with anti-CD68, anti-CD163 or anti-GFAP (Table 1). Followed by the respective secondary antibodies labeled with either goat anti-rabbit Alexa 647 (1:400,Invitrogen) or goat anti-mouse Alexa 488 (Invitrogen). Nuclei were counterstained with 4′-6 diamidino-2-phenylindole (DAPI, 1:25000, Sigma) and sections mounted with Vectashield^®^.

### Statistical analysis

Statistical analyses were performed with IBM SPSS statistics version 20. Statistical significance was set at *P* < 0.05. A non-parametric Wilcoxon Signed Ranks Test was used to assess differences between ligand induced migration and CHIPS inhibition.

## Results

### FPR1 is expressed in all GBM patient samples

**Fig. 1:**
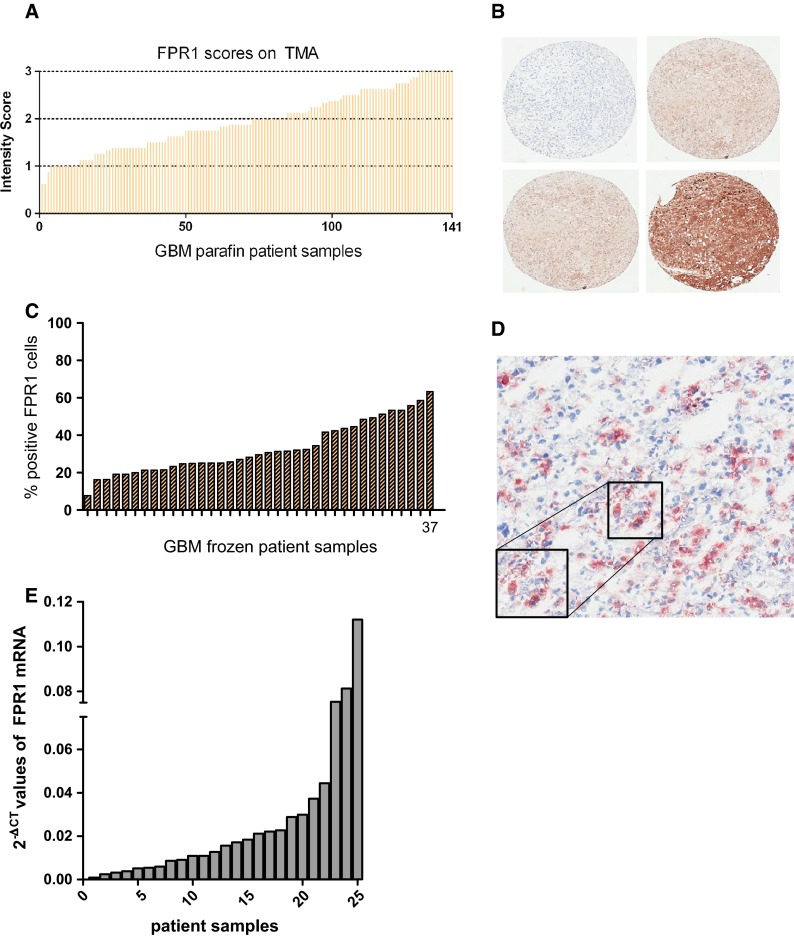
Detection of FPR1 expression on Tissue Micro Array, frozen sections and qPCR. Bar histogram representing results obtained from a Tissue MicroArray (TMA). Immunohistochemical detection of FPR1 (ab#150533) expression on formalin fixed-paraffin embedded GBM patient specimens. Semi-quantitative evaluation was performed by averaging the scores of 4 cores derived from each patient specimen and plotted with a corresponding bar. The intensity of FPR1 expression was scored on a scale from 0 to 3; 0 being negative, 1 positive but with focal and diffuse staining, 2 prevalently focal and more intense staining and 3 exhibiting highly intense focal staining. On average patient samples exhibited an intensity score of 2. All samples were positive for FPR1 (**a**). Representative pictures of 4 different core biopsies containing FPR1 intensities from 0 to 3 (**b**). Immunohistochemical detection of FPR1 (ab#101659) expression on 36 frozen GBM patient samples and quantification containing on average 32 ± 14 % FPR1 positive cells (**c**). Representative FPR1 immunohistochemical staining on a frozen GBM section (**d**). FPR1 mRNA detection on GBM snap frozen tissue samples by qPCR. All samples were loaded in 4 replicates, FPR1 mRNA values varied from minimum (2^−ΔCT^ = 9.34 × 10^−4^) to maximum (2^−ΔCT^ = 1.1 × 10^−1^) (**e**)

Immunohistochemical analysis of GBM samples (FFPE) showed that FPR1 has a relative diffuse cytoplasmic staining in tumor cells. Blood vessels were negative for FPR1 and served as a negative internal control. All 141 patient samples on TMA showed FPR1 expression. Semi-quantitative evaluation of all core biopsies on TMA resulted in an average intensity of 2 (Fig. 1a, b). Six samples displayed 1 negative core and 2 samples contained 2 negative cores. In the remaining specimens all 4 cores stained positive. In 3-control paraffin sections of pneumonia patient samples, FPR1 was highly expressed on both neutrophils and broncho-alveolar epithelial cells as previously described [15, 16] (Supplementary Fig. 1a). In healthy brain tissue, neuron cell bodies were slightly FPR1 positive [17] (Supplementary Fig. 1b). Quantitative evaluation of FPR1 in 37 frozen GBM sections resulted in an average amongst all tumors of 33 ± 14 % of FPR1 positive cells (Fig. 1c, d). In the frozen sections FPR1 expression was stronger along the cell membranes than in the cytoplasm.

FPR1 mRNA was detected in all 25 GBM patient samples. Values varied from the lowest detectable mRNA levels (2^−ΔCT^ = 9.34 × 10^−4^) to the highest detectable levels (2^−ΔCT^ = 1.1 × 10^−1^) (Fig. 1e). These findings indicate that FPR1 is highly expressed in human GBM.

### The activation of U87 cells by mitochondrial peptides can be inhibited with CHIPS

**Fig. 2:**
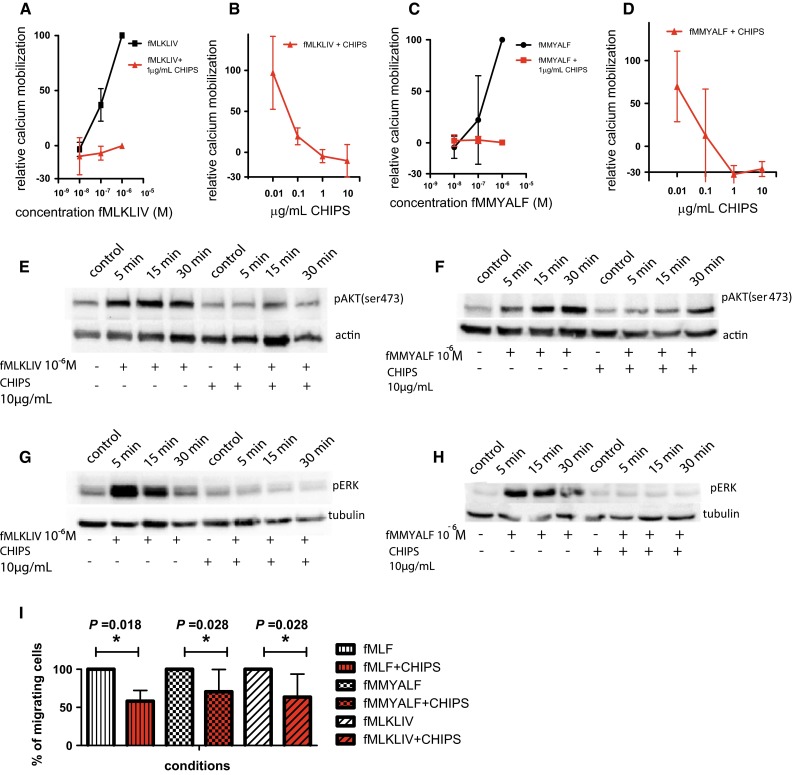
Inhibition of mitochondrial induced FPR1 activity by CHIPS in U87 cells. Mitochondrial peptides fMLKLIV and fMMYALF induce dose dependent calcium mobilization (10^−6^ M–10^−8^ M) of U87 cells (**a**, **c**), which can be dose dependently inhibited with 0.01–10 μg/mL CHIPS (**b**, **d**). In U87 cells 10^−6^ M fMLKLIV or fMMYALF induced AKT phosphorylation on the Ser473 site at 5, 15 and 30 min. At the same time points 10 μg/mL CHIPS showed 46 ± 33, 52 ± 7, 67 ± 12 % inhibition of fMLKLIV induced phosphorylation and respectively 67 ± 39, 78 ± 29, 70 ± 40 % inhibition of fMMYALF-induced phosporylation (**e**, **f**). A concentration of 10^−6^ M fMLKLIV or fMMYALF induce ERK1/2 phosphorylation in U87 cells at 5, 15 and 30 min. When U87 cells were pre-treated with CHIPS (10 μg/mL), fMLKLIV induced phosphorylation was inhibited up to 100 % and fMMYALF-induced phosphorylation was inhibited 94 ± 16, 99 ± 1 and 92 ± 12 % (respectively at time points 5, 15 and 30 min) (**g** and **h**). For migration in transwell assays each time the cell migration towards one of the ligands was set at 100 % and inhibition with CHIPS was plotted against it. Migration towards fMLF, fMMYALF and fMLKLIV of U87 cells was inhibited up to 42 ± 14 % (*P* = 0.018), 34 ± 27 % (*P* = 0.028) and 36 ± 29 % (*P* = 0.028) when pretreated with 10 μg/mL CHIPS. Values are indicated as mean ± SD (**i**)

U87 cells stimulated with fMLKLIV and fMMYALF exhibited a dose dependent calcium release up to 37 ± 15 % (fMLKLIV) and 30 ± 49 % (fMMYALF) (Fig. 2a, c). CHIPS treatment completely inhibited calcium release induced by mitochondrial derived fMLKLIV and fMMYALF (Fig. 2b, d; Table 2). These results show that mitochondrial peptides induce calcium mobilization in U87 cells, which can be inhibited with CHIPS.

**Table 2:** Ligand induced calcium mobilization with CHIPS inhibition of U87 cells

Concentration	Inhibition with CHIPS (%)				
	0 μg/mL % stimulation	0.01 μg/mL	0.1 μg/mL	1 μg/mL	10 μg/mL
fMLKLIV					
10^−6^ M	100	–	–	100 ± 2	–
10^−7^ M	22 ± 43	3.0 ± 34	81 ± 8	105 ± 6	110 ± 15
10^−8^ M	−4.3 ± 7.3	–	–	100 ± 2	–
fMMYALF					
10^−6^ M	100	30 ± 31	87 ± 41	114 ± 25	126 ± 6
10^−7^ M	30 ± 19	–	–	97 ± 3.8	–
10^−8^M	−5.0 ± 6	–	–	98 ± 5.7	–

### CHIPS inhibits mitochondrial peptide induced AKT and ERK1/2 phosphorylation in U87

Stimulation of U87 cells for 5, 15 or 30 min with fMLKLIV or fMMYALF, induced AKT(ser473) phosphorylation (Fig. 2e, f). Densitometric quantification showed that at the same time points, CHIPS pre-treatment inhibited respectively 46 ± 33, 52 ± 7 and 67 ± 12 % of the fMLKLIV-induced phosphorylation (Supplementary Fig. 2a). Similarly in cells stimulated with fMMYALF and pre-treated with CHIPS, respectively 67 ± 39, 78 ± 29 and 70 ± 40 % of fMMYALF-induced AKT phosphorylation was inhibited (Supplementary Fig. 2b). Furthermore fMLKLIV-induced phosporylation of ERK1/2 at 5, 15 and 30 min was completely inhibited with CHIPS (100 %) at all three time points (Fig. 2g) (Supplementary Fig. 2c). Moreover at 5, 15, and 30 min, fMMYALF-induced ERK1/2 phosphorylation could be inhibited up to respectively 94 ± 16, 99 ± 1 and 92 ± 12 % when treated with CHIPS (Fig. 2h)(Supplementary Fig. 2d). This indicates that mitochondrial peptide induced phosphorylation of AKT and ERK1/2 in U87 cells can be potently inhibited with CHIPS.

### CHIPS inhibits U87 migration triggered by fMLKLIV, fMMYALF and fMLF

U87 cells migrated towards the ligands fMLF, fMMYALF and fMLKIV. When preincubating cells with 10 µg/mL CHIPS, cell migration towards fMLF (control), fMMYALF and fMLKLIV was inhibited up to 42 ± 14 % (*P* = 0.018), 34 ± 27 % (*P* = 0.028) and 36 ± 29 % (*P* = 0.028) respectively (Fig. 2i), indicating that migration of U87 cells towards mitochondrial peptides can be inhibited with CHIPS.

### FPR1 activation and mRNA expression could not be detected in GG cell lines

None of the GG cell lines showed calcium mobilization upon stimulation with fMLF (Supplementary Fig. 3). Additionally FPR1 mRNA could not be detected in any of the 8 GG cell lines while the U87 positive control did exhibit FPR1 mRNA (2^−ΔCT^ = 2.3 × 10^−3^)(Supplementary Fig. 2e). These results indicate that GG cell lines in vitro retained no functional FPR1 and did not express FPR1 mRNA.2

### FPR1 is expressed on primary tumor tissue from which GG cell lines were isolated

**Fig. 3:**
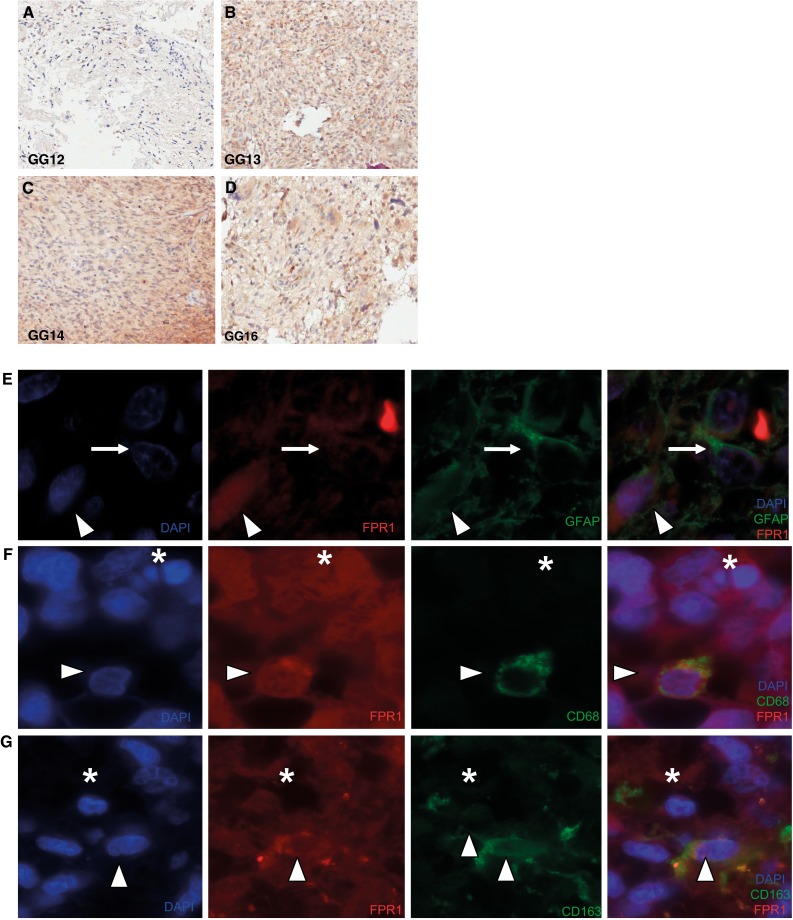
Immuno -hisotochemical and -fluorescence detection of FPR1 (ab#150533) alone or coupled with GFAP and CD68/CD163 markers. Representative photomicrographs depicting immunohistochemical expression of FPR1 in the GBM patient specimens and its derived primary GBM cell line. The original patient specimens all displayed FPR1 expression with variable intensities. Images of patient GBM tissue of which GG cell lines were obtained (**a**–**d**). Immunofluorescence image displaying GFAP single expression (*arrow*) and co-expression with FPR1 (*arrow head*) (*panel* **e**). FPR1 shows single expression (*asterisk*) and co-expression with CD68 (*arrow head*) (*panel* **f**). Single stained FPR1 cells (*asterisk*) and co-expression with CD163 (*arrow head*) (*panel* **g**)

The discrepancy between FPR1 expression in GBM patient samples (Fig. 1a–d) and its absence in GG cell lines prompted us to evaluate the primary GBM patient material from which these cells were isolated. Interestingly immunohistochemistry on the paired tumors from which the GG cell lines were originally obtained, all exhibited FPR1 positivity (Fig. 3a–d, Supplementary Fig. 4).

### In GBM tissue samples FPR1 is expressed on glial tumor cells and on macrophages

FPR1 is primarily known for its expression on immune cells [18]. To confirm the presence of FPR1 on tumor cells, double immunofluorescence was performed on GBM patient samples. Results showed FPR1 co-expression with GFAP (Fig. 3e), and also with CD68 and CD163 (Fig. 3f, g). This indicates that FPR1 expression is present on tumor cells as well as on macrophages.

### FPR1 expression is present in orthotopic tumors of early passage glioblastoma cells

**Fig. 4:**
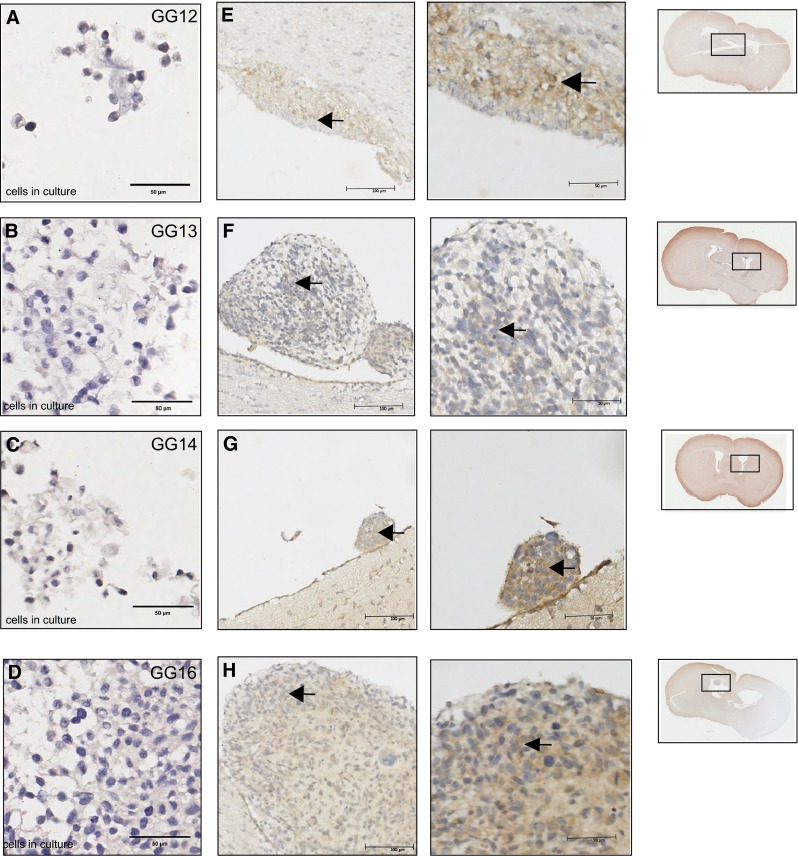
Immunohistochemical detection of FPR1 (ab#150533) expression in brain tissue sections of NOD-SCID IL-2 γ-knockout mice orthotopically injected with GG cell lines. Paraffin embedded samples of the cell lines GG12, GG13, GG14 and GG16 were negative for FPR1 (**a**–**d**). Mouse brain injected with GG12, GG13, GG14 and GG16 cell lines all showed tumor formation. All tumors expressed FPR1 with variable intensities (*panels* **e**–**h**). *Arrow heads* indicate the FPR1 positively stained GG cells

The GG cell lines tested were FPR1 negative in vitro, while the paired GBM tissues expressed FPR1 (Fig. 3a–d, Supplementary Fig. 4). Therefore the tumor tissue obtained by orthotopic implantation in the brain of GG cell lines in NOD-SCID IL-2 γ-knockout mice was evaluated. Tumors were positive with variable intensities (Fig. 4e–h), while all slides from FFPE blocks of cultured GG cell lines were FPR1 negative (Fig. 4a–d). Negative controls are depicted Supplementary Fig. 5. The human nature of the tumors was confirmed with anti-human-nestin staining [12, 19]. These findings suggest that GG cell lines lacking FPR1 in vitro, form tumors with regained receptor expression when intracranially implanted in NOD-SCID IL-2 γ-knockout mice.

## Discussion

Our findings show that the FPR1 protein is highly expressed in GBM and that its expression is stimulated by the microenvironment. We detected FPR1 expression in a large series of human GBM tumors and showed that the migration of U87 cells is activated by human mitochondrial peptides. Mitochondrial peptide induced activity in U87 cells could be inhibited with CHIPS. FPR1 expression was not detected in 8 early passage cell lines isolated from primary GBM tissue, while their originating tumors did express FPR1. When these cell lines were intracranially injected in mice, the developed tumors regained FPR1 expression thus suggesting a role for the microenvironment in fostering the expression of FPR1.

This is the first time that immunohistochemical FPR1 expression has been investigated in a large series of human GBM tumors. FPR1 immunohistochemistry showed differences in staining patterns for paraffin and frozen human GBM sections. Specifically paraffin sections exhibited a more diffuse cytoplasmic staining while the frozen sections showed stronger membranous staining. This is likely due to differences between the two antibodies used; one being directed against the second extracellular loop and the other directed against the internal region of FPR1. The only other study investigating FPR1 in six GBM patient specimens reported receptor expression in all GBM specimens as detected by immunohistochemistry [6]. Overall our findings of the universal expression of FPR1 in human GBM tumors, together with previous reports on the contribution of FPR1 to the survival benefit of animals treated with siRNA against FPR1 [9, 20], indicate that this receptor might be a interesting target for novel drug development.

GBMs are typically characterized by extensive areas of necrosis. Previous findings showed that the supernatant of necrotic U87 cells activated FPR1 as proven by calcium mobilization and increased chemotaxis [6]. The effects of the necrotic supernatant on FPR1 were probably, at least in part, caused by the presence of free-floating mitochondrial peptides. Mitochondrial peptide affinity and activity on FPR1 was previously reported in transfected HL-60 cells [21]. Together with bacterial peptide fMLF these are the only source formylated peptides in nature and are characterized by highly conserved patterns. This indicates that the ruptured cells in the necrotic GBM microenvironment may affect the broad number of FPR1 positive cells that are present.

We showed for the first time that mitochondrial peptides directly affect the behavior of U87 cells. Activation of the FPR1 expressing U87 GBM cell line by mitochondrial peptides fMLKLIV and fMMYALF elicited calcium mobilization, FPR1 downstream protein phosphorylation and migration. All these responses could be inhibited with CHIPS. Our results are in line with previous findings, which showed a role for FPR1 in malignant tumor cell activity. Namely, incubation of U87 with the bacterial peptide fMLF stimulated cell migration [6], induced the upregulation of HIF-1α [6] and VEGF [6, 9]. However the use of mitochondrial peptides resembles more closely the conditions of the tumor milieu. Moreover the ability of CHIPS to inhibit all mitochondrial peptide induced responses on U87 cells makes it a potential drug for further investigation.

To further study the effects of the microenvironment on FPR1 we screened a number of early passage GBM cell lines for FPR1 expression. Early passage GBM cell line models are often used to bring new insights into the etiology of GBM [25] and are generally considered to resemble the primary tumor more closely than established cell lines [26]. The observation that none of the 8 tested GG cell lines expressed FPR1 was quite unexpected, as all the paired GBM samples did express FPR1. This discrepancy could be the result of heterogeneous FPR1 expression within the tumor or of FPR1 being mostly present on immune cells. In original tumor material, with double immunofluorescence we confirmed that receptor expression occurred in GFAP-positive astrocytic tumor cells as well as on CD68/CD163-positive microglia/macrophages. Huang and colleagues found no co-expression of FPR1 and GFAP, which led them to conclude that FPR1 is associated with a more undifferentiated cell state [27]. These experiments were performed on U87 cells and thus in an in vitro setting. The GG cell lines were cultured as neurospheres in serum free media supplemented with EGF and FGF, favoring the growth of undifferentiated, cancer stem-like cells [12]. However, we could not detect FPR1 in our early passage GG cell lines. Next we showed that when implanted into mouse brains, the GG cells lacking FPR1 expression in vitro, exhibited the FPR1 membrane expression in vivo. These results show that the GG cell lines still retain the capacity to express FPR1 and suggest that the microenvironment influences the receptor expression of these cells.

Overall, FPR1 is highly expressed in GBMs and the microenvironment plays an important role in modulating FPR1 activation and expression.

## Electronic supplementary material

Supplementary material 1 (DOCX 5304 kb)

Supplementary material 2 (PDF 4257 kb)

Supplementary material 3 (PDF 356 kb)

Supplementary material 4 (PDF 1561 kb)

Supplementary material 5 (PDF 21000 kb)

Supplementary material 6 (PDF 7460 kb)
