# Supplementary material for: Microenvironment involved in FPR1 expression by human glioblastomas
Source: J Neurooncol. 2015 Apr 19;123(1):53–63. doi: 10.1007/s11060-015-1777-2 (PMC4439437; doi:10.1007/s11060-015-1777-2)
Supplement: Supplementary file 1 — Supplementary material 1 (DOCX 5304 kb) [file 11060_2015_1777_MOESM1_ESM.docx]

**Supplementary material**

**Supplementary Fig 1.** Representative specimen of pneumonia patient sample immunohistochemically stained for FPR1. FPR1 is highly expressed on neutrophils and broncho-alveolar cells **(a)**. Specimen of healthy brain tissue immunohistochemically stained for FPR1. Neuron cell bodies exhibit slight positivity for FPR1 **(b)**.

**Supplementary Fig 2.** Densitometric values of Western blot bands of U87 cells stimulated with fMLKIV or fMMYALF. CHIPS could inhibit fMLKLIV induced pAKT **(a)** and fMMYALF induced pAKT **(b)**. Accordingly CHIPS could inhibit fMLKLIV induced pERK1/2 **(c)** and fMMYALF induced pERK1/2 **(d)**. Detection of FPR1 mRNA levels in positive control cell line U87 but not in GG cell lines **(e)**.

**
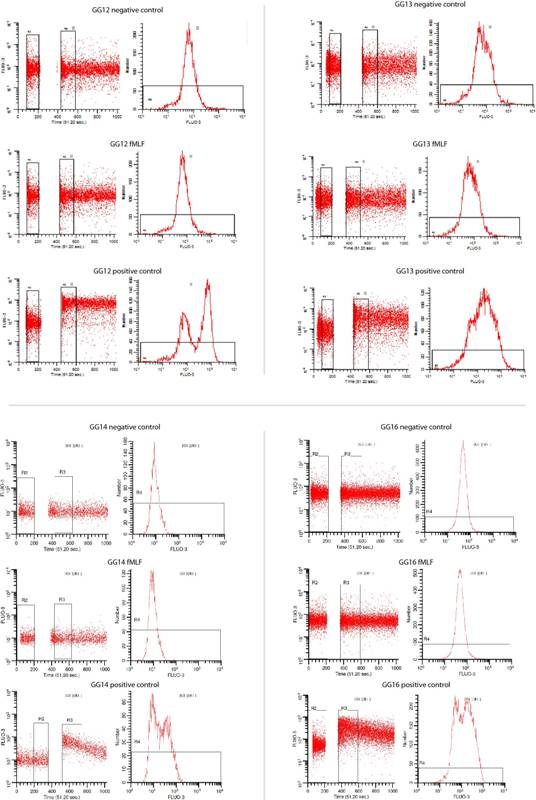
**

**Supplementary Fig 3.** Raw data of Calcium mobilization assays. GG12 **(a)**, GG13 **(b)**, GG14 **(c)** and GG16 **(d)** were each time stimulated with buffer (negative control), fMLF or ionomcyn (positive control). Calcium release could only be measured when applying ionomycin which induces the release of all intracellular calcium labeled ions.

**
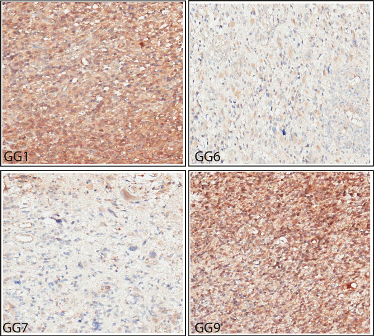
**

**Supplementary Fig 4.** Human GBM specimens immunohistochemically stained for FPR1 all showed positivity. The GG numbers on the bottom-left of each micrograph represent the name of the respective cell line obtained from each GBM tissue sample. Specimens exhibit variable staining intensity.

**Supplementary Fig 5.** Negative controls of GG cell lines. GG12, 13, 14, 16 were tested with appropriate IgG control **(panel a)** and with omission of primary antibody **(panel b)**.
