# Supplementary figures and images for: Microenvironment involved in FPR1 expression by human glioblastomas
Source: J Neurooncol. 2015 Apr 19;123(1):53–63. doi: 10.1007/s11060-015-1777-2 (PMC4439437; doi:10.1007/s11060-015-1777-2)

A

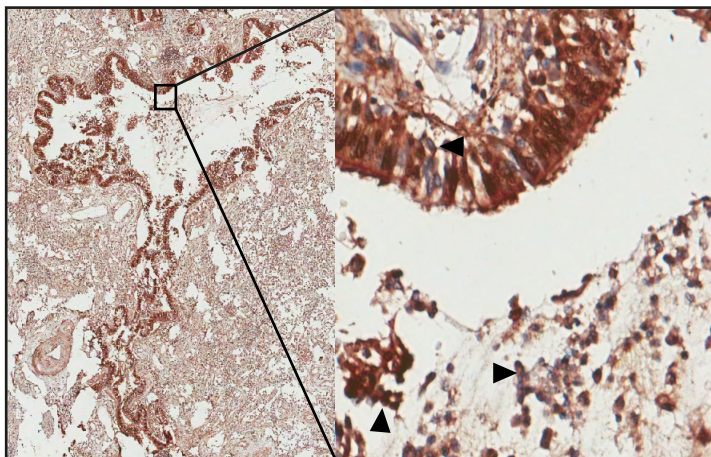

B

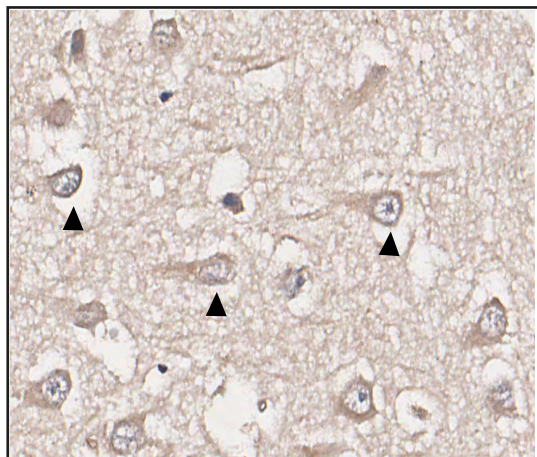

Supplement: Supplementary file 2 — Supplementary material 2 (PDF 4257 kb) [file 11060_2015_1777_MOESM2_ESM.pdf]

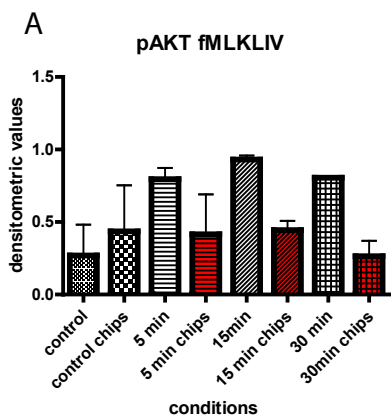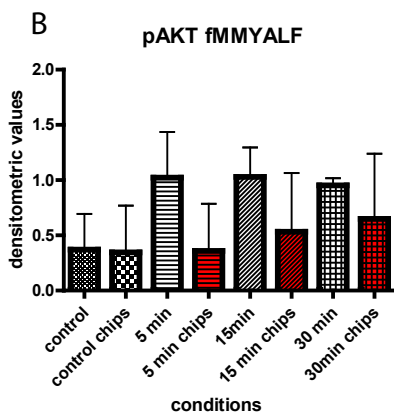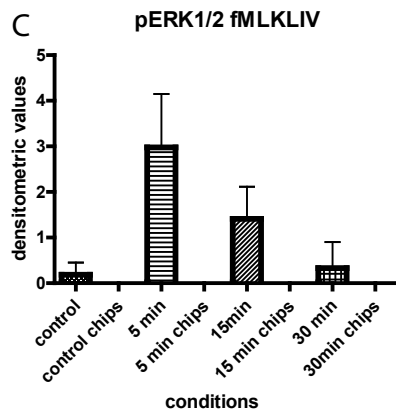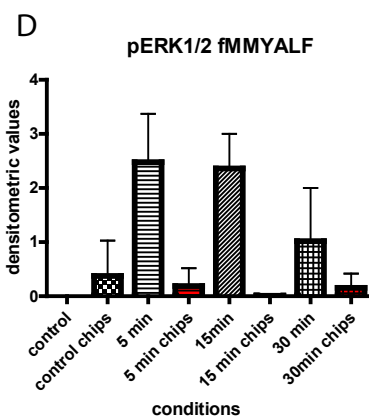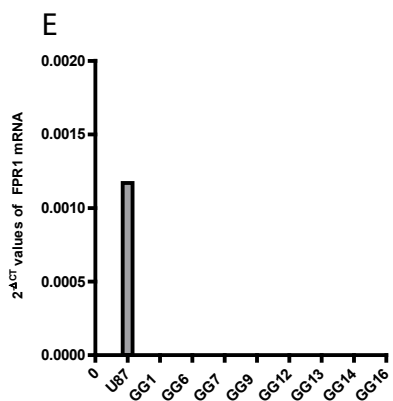

Supplement: Supplementary file 3 — Supplementary material 3 (PDF 356 kb) [file 11060_2015_1777_MOESM3_ESM.pdf]

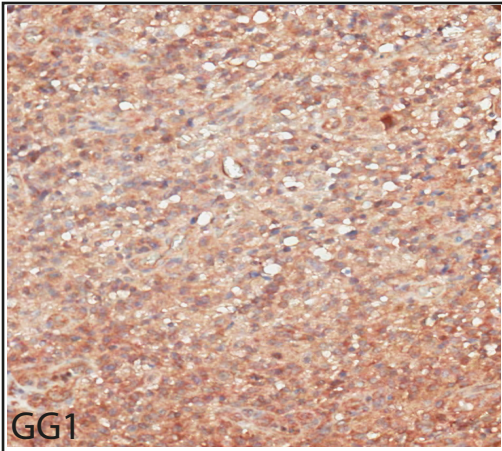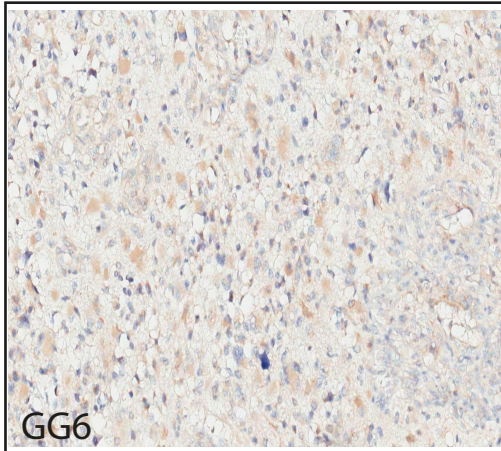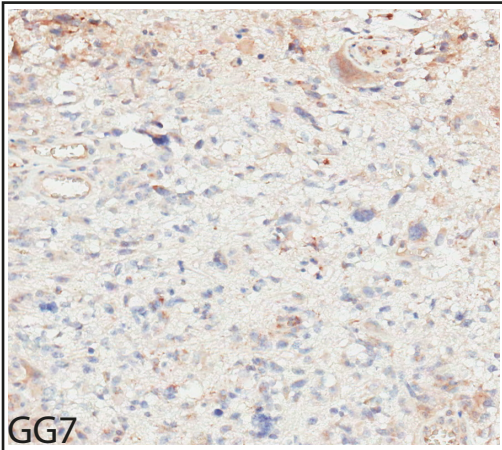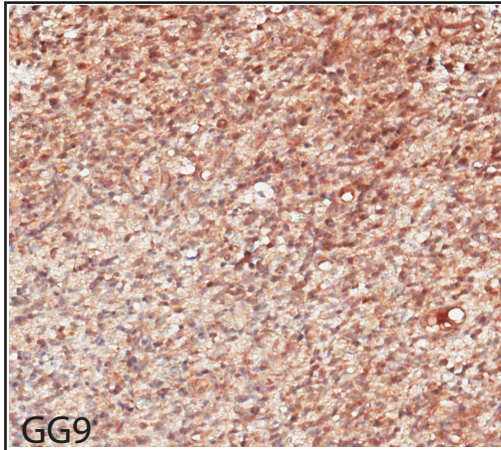

Supplement: Supplementary file 5 — Supplementary material 5 (PDF 21000 kb) [file 11060_2015_1777_MOESM5_ESM.pdf]
