# Supplementary material for: Microenvironment involved in FPR1 expression by human glioblastomas
Source: J Neurooncol. 2015 Apr 19;123(1):53–63. doi: 10.1007/s11060-015-1777-2 (PMC4439437; doi:10.1007/s11060-015-1777-2)
Supplement: Supplementary file 4 — Supplementary material 4 (PDF 1561 kb) [file 11060_2015_1777_MOESM4_ESM.pdf]

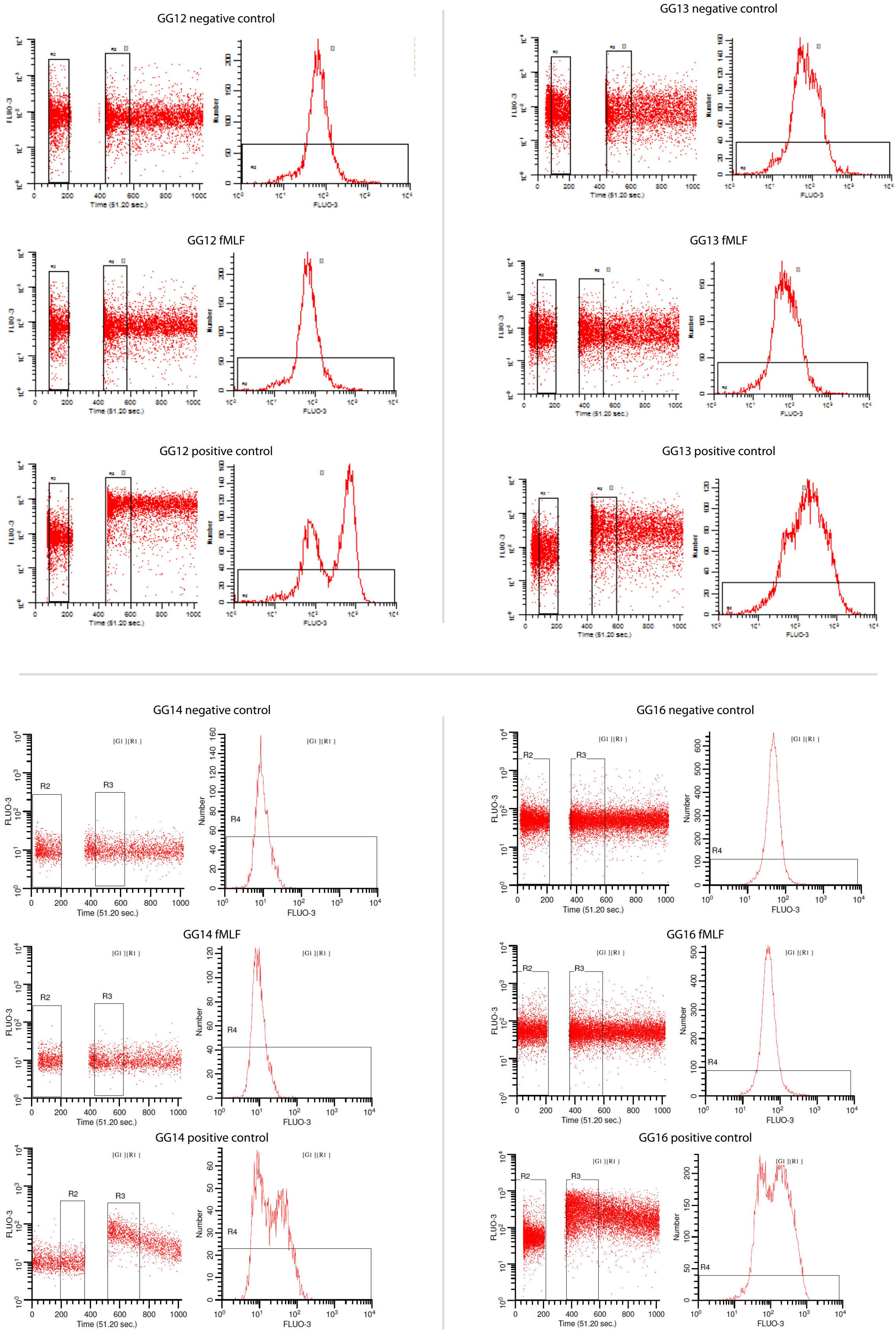
GG12 negative control
GG13 negative control
GG12 fMLF
GG13 fMLF
GG12 positive control
GG13 positive control
GG14 negative control
GG16 negative control
GG14 fMLF
GG16 fMLF
GG14 positive control
GG16 positive control
FLUO-3
Time (51.20 sec.)
Number
R2
R3
R4
[G1][R1]
