# Supplementary material for: Microenvironment involved in FPR1 expression by human glioblastomas
Source: J Neurooncol. 2015 Apr 19;123(1):53–63. doi: 10.1007/s11060-015-1777-2 (PMC4439437; doi:10.1007/s11060-015-1777-2)
Supplement: Supplementary file 6 — Supplementary material 6 (PDF 7460 kb) [file 11060_2015_1777_MOESM6_ESM.pdf]

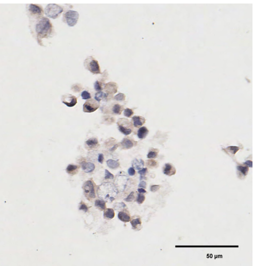

GG12 IgG control

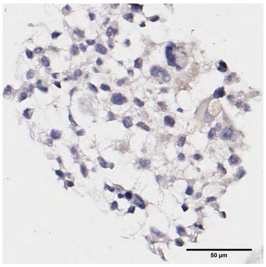

GG13 IgG control

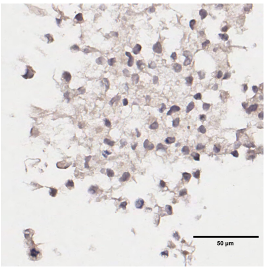

GG14 IgG control

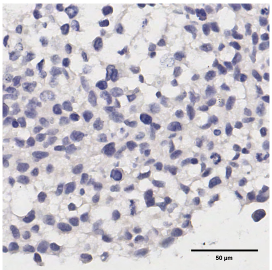

GG16 IgG control

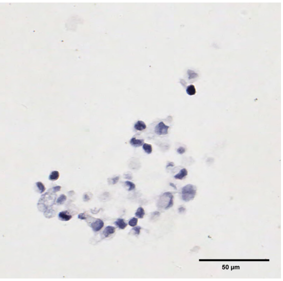

GG12 PBS control

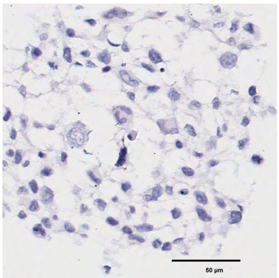

GG13 PBS control

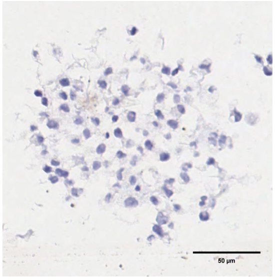

GG14 PBS control

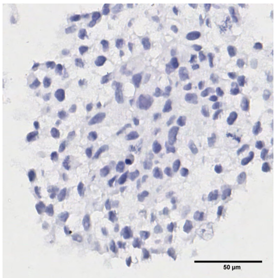

GG16 PBS control
